# PhosphoLIMBO: An Easy and Efficient Protocol to Separate and Analyze Phospholipids by HPTLC From Plant Material

**DOI:** 10.21769/BioProtoc.5434

**Published:** 2025-09-05

**Authors:** Louise Fougère, Hortense Moreau, Cécile Mirande-Bret, Laetitia Fouillen, Yohann Boutté

**Affiliations:** Univ. Bordeaux, CNRS, Laboratoire de Biogenèse Membranaire, UMR 5200, F-3314015 Villenave d’Ornon, France;

**Keywords:** Plant biology, Phospholipids, Anionic phospholipids, Biological membrane, Lipid extraction, HPTLC, Lipidomic

## Abstract

Phospholipids are major structural and regulatory elements of biological membranes and are involved in many different cellular and physiological processes. In this protocol, we provide an easy, cost-effective, and efficient method to obtain an overview of the phospholipid composition using high-performance thin layer chromatography (HPTLC). While the currently known phospholipid separation methods based on HPTLC display co-migration of certain lipid classes, the method we describe here allows the separation of all phospholipid classes, including anionic phospholipids in plant samples. This protocol combines elements of the classical Vitiello and Touchstone solvent systems to optimize phospholipid separation in a scaled pattern. Here, we provide a full characterization of this method, including statistical analyses of the retention factor of each phospholipid to show the robustness of the method and its efficiency in separating all phospholipid classes of a biological sample.

Key features

• Analysis of phospholipid composition through an easy, fast, robust, and cost-effective HPTLC method.

• Separation of anionic phospholipids from the other phospholipid species.

• Full overview of all phospholipids categories, including anionic phospholipids.

• Qualitative and quantitative approach.

## Graphical overview


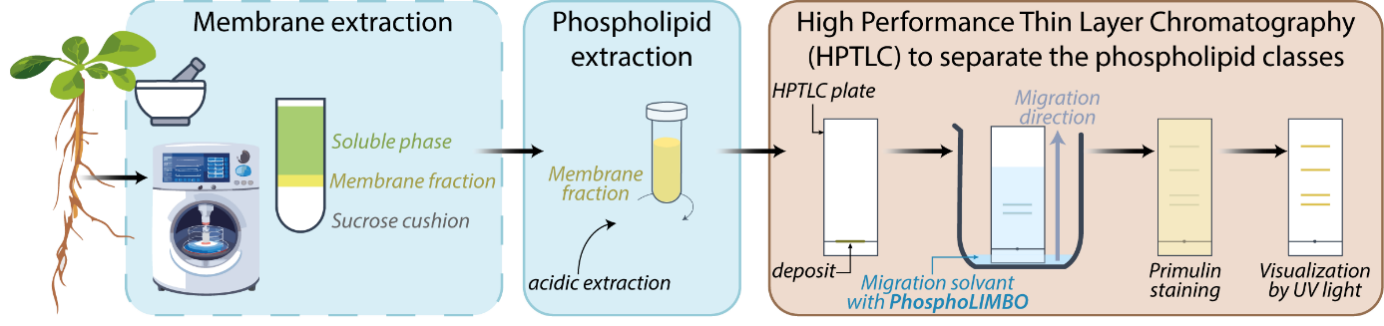


## Background

Lipids are major molecular and cellular actors due to the structural properties they confer to cellular membranes and their functional roles in, but not limited to, cell signaling, intracellular trafficking, cell division, cell growth, and differentiation [1]. Lipids are classified into different classes according to their chemical structure, of which three main classes are predominant: glycerolipids, sphingolipids, and sterols. Glycerolipids are the most abundant class of lipids in plants and comprise diacylglycerol (DAG), triacylglycerols (TAG), glycolipids such as galactolipids [e.g., monogalactosyldiacylglycerol (MGDG), digalactosyldiacylglycerol (DGDG), and the sulpholipid sulfoquinovosyl diacylglycerol (SQDG)], and phosphoglycerides, commonly named phospholipids. Depending on the nature of the hydrophilic group attached to the phosphate, phospholipids are ranked into different categories: neutral, as phosphatidylglycerol (PG), phosphatidylethanolamine (PE) and phosphatidylcholine (PC), or anionic phospholipids, as phosphatidic acid (PA), phosphatidylserine (PS), and phosphadylinositol (PI) and its phosphate derivatives phosphatidylinositol-phosphate (PIPs) and phosphatidylinositol-biphosphate (PIP_2_) [2]. PC is the most abundant phospholipid [around 20% in the plant plasma membrane (PM)]. At the PM, anionic phospholipids are less prominent, with PI and PS each representing around 5%, and PIPs and PIP2 representing less than 1% [3]. Glycerolipids have been shown to be involved in a wide range of physiological processes in plants. Glycolipids are important for photosynthesis, early plant development before photosynthesis establishes (lipid droplets in seeds), and resistance to environmental stresses such as phosphate starvation [4,5]. Phospholipids are important structural elements of the membranes; anionic phospholipids, despite their low abundance, have been shown to be crucial lipids to cell signaling, polar trafficking, autophagy, cell division, and cell–cell communication [1,6–10]. Thus, the detection and quantification of glycerolipids, including glycolipids, phospholipids, and anionic phospholipids, is crucial in lipid research.

Protocols for the detection and quantification of glycerolipids by liquid chromatography coupled to mass spectrometry (LC–MS/MS) have been deployed previously [11,12]. However, the accessibility of LC–MS/MS equipment and the expertise required to run and interpret mass-spectrometry results might be a bottleneck. High-performance thin layer chromatography (HPTLC) allows the separation of phospholipids from a lipid extract. HPTLC is an accessible, fast, and cost-effective method. A widely used method to detect phosphoglycerolipids is based on the radioactive element ^32^Pi to maximize sensitivity, allowing quantification of anionic phospholipids [13]. Radioactive-free methods exist and are commonly based on Vitiello and Touchstone protocols [14,15]. However, these methods have some limitations. PA/PG are not separated using the Vitiello solvent mix, while PE/PI are not separated using the Touchstone method. In the same way, in yeast, the solvent mix classically used cannot separate PI/PS [16,17]. The same issue was reported in murine skin melanoma cells [18]. In addition, in all these methods, PIPs and PIP_2_ are stuck close to the deposit line and are not mobilized during migration [14,15]. In this protocol, we optimize a solvent mix that we called PhosphoLIMBO (for PhosphoLIpid Migration Best Optimization and in reference to the limbo dance, where the goal is to pass under a scale). This protocol enables the separation in a scaled pattern of all species of glycerolipids extracted from a microsomal fraction of *Arabidopsis thaliana* seedlings. Our method is based on a combination of several published protocols [14,15], is radioactive-free, easy, and cost-effective to implement, and has already been successfully used in several publications [19,3].

## Materials and reagents

**Biological materials**

1. Columbia-0 (Col-0) seeds (NASC, catalog number: CS1093)

**Reagents**

1. Bleach (Manutan, catalog number: LJ26659A)

2. Murashige and Skoog powder (Duchefa Biochemie, catalog number: M0222.0050)

3. HEPES (Sigma-Aldrich, catalog number: H4034-500G)

4. Potassium hydroxide (KOH) (Sigma-Aldrich, catalog number: P5958-500G)

5. Sucrose (Sigma-Aldrich, catalog number: 84100-1KG)

6. Magnesium chloride (MgCl_2_) (Sigma-Aldrich, catalog number: 208337-100G)

7. Dithiothreitol (Euromedex, catalog number: EU0006-D)

8. Polyvinylpyrrolidone (Sigma-Aldrich, catalog number: P2307-100G)

9. Phenylmethylsulfonyl fluoride (PMSF) (Roche, catalog number: 10837091001)

10. Dimethyl sulfoxide (DMSO) (Sigma-Aldrich, catalog number: D8418-250ML)

11. Ethylenediaminetetraacetic acid (EDTA) (Sigma-Aldrich, catalog number: ED4SS-1KG)

12. Cocktail protease inhibitor (Sigma-Aldrich, catalog number: P9599-5ML)

13. Chloroform (Fisher Chemical, catalog number: C/4960/17)

14. Methanol (Fisher Chemical, catalog number: M/4062/17)

15. Hydrochloric acid (HCl) (Sigma-Aldrich, catalog number: 320331-2.5L)

16. 2-Propanol (Sigma-Aldrich, catalog number: 33539-2.5L-M)

17. Potassium chloride (KCl) (Sigma-Aldrich, catalog number: P9333-500G)

18. Methyl acetate (Acros Organics, catalog number: 181380025)

19. Triethylamine (Acros Organics, catalog number: 157910010)

20. HPTLC Silica gel 60 F254 20 × 10 cm (Sigma-Aldrich, catalog number: 1.05642.0001)

21. Sodium chloride (NaCl) (Euromedex, catalog number: 1112-A)

22. Potassium phosphate monobasique (KH_2_PO_4_) (Sigma-Aldrich, catalog number: P5655-500G)

23. Di-sodium hydrogen phosphate (Na_2_HPO_4_) (Euromedex, catalog number: 1309)

24. Brain phosphatidylinositol-4,5-biphosphate (PI4,5P_2_) (Avanti polar lipid, catalog number: 850155P)

25. 18:1 phosphatidylinositol-4-phosphate (PI4P) (Avanti polar lipid, catalog number: 850151P)

26. 18:1 phosphatidylinositol-3-phosphate (PI3P) (Avanti polar lipid, catalog number: 850150P)

27. Soybean phosphatidylinositol (PI) (Sigma-Aldrich, catalog number: 79401)

28. Soybean phosphatidylserine (PS) (Sigma-Aldrich, catalog number: P0474)

29. Egg yolk phosphatidylcholine (PC) (Sigma-Aldrich, catalog number: P3556)

30. Egg yolk phosphatidic acid (PA) (Sigma-Aldrich, catalog number: P9511)

31. Egg yolk phosphatidyl ethanolamine (PE) (Sigma-Aldrich, catalog number: P6386)

32. Egg yolk lecithin phosphatidyl glycerol (PG) (Sigma-Aldrich, catalog number: P8318)

33. β-Sitosterol (Avanti polar lipid, catalog number: 700095P)

34. Plant MGDG (Avanti polar lipid, catalog number: 840523)

35. Plant DGDG (Avanti polar lipid, catalog number: 840524)

36. 20:0 MG (NU-CHEK PREP, catalog number: M-174)

37. 18:1 DG (Avanti polar lipid, catalog number: 800811)

38. 1,3-Dioleoyl-2-palmitoylglycerol (TG) (Sigma-Aldrich, catalog number: D1657)

39. MilliQ water (H_2_O) (sterile and non sterile)

40. Acetone (Sigma-Aldrich, catalog number: 32201-2.5L-M)

41. Primulin (Sigma-Aldrich, catalog number: 206865-5G)

**Solutions**

1. HEPES solution (see Recipes)

2. 2 M MgCl_2_ (see Recipes)

3. 1 M Dithiothreitol (see Recipes)

4. Membrane extraction buffer (see Recipes)

5. PMSF solution (see Recipes)

6. 38% sucrose solution (see Recipes)

7. Membrane resuspension buffer (see Recipes)

8. 0.25% KCl (see Recipes)

9. 2 M HCl (see Recipes)

10. 1 M HCl (see Recipes)

11. 0.01 M HCl (see Recipes)

12. Phospholipid extraction “Kill” mix (see Recipes)

13. Phospholipid wash mix (see Recipes)

14. 10× PBS (see Recipes)

15. Migration solvent mix, PhosphoLIMBO (see Recipes)

16. Primulin solution (see Recipes)

**Recipes**

*Note: For safety reasons, we recommend using solvents under an extractor hood and wearing personal protective equipment: gloves, lab coat, and safety goggles. When using acids such as HCl, always add the acid to the water with the same safety measures as for solvents.*

**1. HEPES solution**

**Table:** 

Reagent	Final concentration	Amount
HEPES	50 mM	12 g
H_2_O	n/a	1,000 mL

Adjust pH to 7.5 with KOH.

*Note: Can be stored at 4 °C for a few months.*

**2. 2 M MgCl_2_**

**Table:** 

Reagent	Final concentration	Amount
MgCl_2_	2 M	9.52 g
H_2_O	n/a	50 mL

*Note: The MgCl_2_ powder must be added very cautiously inside glass containers (due to exothermic reaction in water).*

**3. 1 M Dithiothreitol**

**Table:** 

Reagent	Final concentration	Amount
Dithiothreitol	1 M	7.712 g
H_2_O	n/a	50 mL

*Note: Can be stored at -20 °C for a few months.*

**4. Membrane extraction buffer**

**Table:** 

Reagent	Final concentration	Amount
Sucrose	0.45 M	77 g
MgCl_2_	5 mM	1.25 mL (from 2 M stock solution)
Dithiothreitol	1 mM	500 μL (from 1 M stock solution)
Polyvinylpyrrolidone	0.5 g/100 mL	2.5 g
HEPES (50 mM, pH 7.5)	n/a	500 mL

*Note: Can be stored at -20 °C for a few months.*

**5. PMSF solution**

**Table:** 

Reagent	Final concentration	Amount
PMSF	100 mM	0.174 g
DMSO	n/a	10 mL

PMSF 1 mM needs to be prepared freshly in the membrane extraction buffer.

*Note: Can be stored at -20 °C for a few months.*

**6. 38% sucrose solution**

**Table:** 

Reagent	Final concentration	Amount
Sucrose	38 g/100 mL	38 g
HEPES (50 mM, pH 7.5)	n/a	100 mL

*Note: Can be stored at -20 °C for a few months.*

**7. Membrane resuspension buffer**

**Table:** 

Reagent	Final concentration	Amount
Sucrose	0.25 M	0.34 g
MgCl_2_ (2 M)	1.5 mM	3 μL
EDTA (0.5 M, pH8)	0.2 mM	1.6 μL
NaCl (5 M)	150 mM	120 μL
HEPES (50 mM, pH 7.5)	n/a	4 mL
PMSF	1 mM	40 μL
Cocktail Protease Inhibitor	1 mL/100 mL	40 μL

*Note: PMSF and cocktail protease inhibitor must be added at the last moment and kept on ice.*

**8. 0.25% KCl**

**Table:** 

Reagent	% solution	Amount
KCl	0.25%	0.25 g
Water	n/a	100 mL

**9. 2 M HCl**

**Table:** 

Reagent	Final concentration	Amount
HCl (37% fumant)	2 M	9.86 mL
Water	n/a	30.14 mL

**10. 1 M HCl**

**Table:** 

Reagent	Final concentration	Amount
HCl (2 M)	1 M	25 mL
Water	n/a	50 mL

**11. 0.01 M HCl**

**Table:** 

Reagent	Final concentration	Amount
HCl (1 M)	0.01 M	500 μL
Water	n/a	50 mL

**12. Phospholipid extraction “Kill” mix**

**Table:** 

Reagent	% solution	Amount
Methanol	64.57%	484 mL
Chloroform	32.29%	242 mL
HCl (1 M)	3.14%	24 mL
Total	n/a	750 mL

Shake vigorously by hand.

**13. Phospholipid wash mix**

**Table:** 

Reagent	% solution	Amount
Methanol	26.66%	12 mL
Chloroform	53.34%	24 mL
HCl (0.01 M)	20%	9 mL
Total	n/a	45 mL

Shake vigorously by hand. Let the solution stabilize for a few minutes in a two-phase solution.

**14. 10× PBS**

**Table:** 

Reagent	Final concentration	Amount
Na_2_HPO_4_·2H_2_O	2.05 g/L	2.05 g
KH_2_PO_4_	240 mg/L	0.24 g
NaCl	800 mg/L	0.8 g
KCl	200 mg/L	0.2 g
Water	n/a	1,000 mL

Adjust pH at 7.4. Shake vigorously by hand.

*Note: Can be stored at room temperature on the bench.*

**15. Migration solvent mix, PhosphoLIMBO**

**Table:** 

Reagent	Final concentration	Amount
Chloroform	30.3%	5 mL
Methanol	10.4%	1.7 mL
2-propanol	24.2%	4. mL
KCl (0.25%)	7.9%	1.3 mL
Methyl acetate	24.2%	4 mL
Triethylamine	3%	0.5 mL

*Note: We recommend preparing it freshly.*

**16. Primulin solution**

**Table:** 

Reagent	Final concentration	Amount
Primulin	0.1 g/L	20 mg
Acetone	50%	100 mL
PBS (10×)	1×	20 mL
Water	n/a	80 mL

Shake vigorously by hand and let it sit for some minutes on the bench.

*Note: We recommend preparing it freshly.*

**Laboratory supplies**

1. Microtube SafeSeal 1.5 mL (Sarstedt, catalog number: 72.706.400)

2. Microtube SafeSeal, 2 mL, PP, Biosphere^®^ plus (Sarstedt, catalog number: 72.695.200)

3. Falcon 50 (Sarstedt, catalog number: 62.547.254)

4. Falcon 15 (Sarstedt, catalog number: 62.554.502)

5. Erlenmeyer flask Duran^®^ with baffles, GL 45, with 4 baffles, with membrane cap and PP pouring ring (Duran, catalog number: WD21283445)

6. Mortar Haldenwanger^TM^ L 55/3/GLASIERT (Fisher Scientific, catalog number: 10053504)

7. Pestle Haldenwanger^TM^ L 56/00/RAUH (Fischer Scientific, catalog number: 10405011)

8. Short stem funnel (Duran, catalog number: DWK213514103-10EA)

9. Gauze Miracloth (Millipore, catalog number: 475855)

10. 2 mL HPLC vials (Agilent, catalog number: 5182-0714)

11. Pasteur glass pipette (Fisherbrand^TM^, catalog number: 11566963)

12. CAMAG^®^ twin trough chamber for 20 × 10 cm plates, with stainless steel lid (CAMAG, catalog number: 022.5254)

13. FLACON 50 mL SVL 25 (Pyrex, catalog number: 203924)

14. Glass recipient (large enough to hold the plate) (Pyrex, catalog number: 234B000)

## Equipment

1. Autoclave PBI Stematic III (GEMINI, catalog number: 05523)

2. Table shaker (Infors HT, Orbitron)

3. Balances (Sartorius)

4. Scientific Industries SI^TM^ Vortex-Genie^TM^ 2 (Fisher Scientific, catalog number: 15547335)

5. Centrifuge for Falcon50 (Beckman Coulter, Allegra21R)

6. Centrifuge for Eppendorfs (Eppendorf^®^, Centrifuge 5425 G, catalog number: EP5405000760-1EA)

7. Ultracentrifuge (Thermo Scientific^TM^, Sorvall^TM^ WX+, catalog number: 15362177)

8. Ultracentrifuge rotors for 12 mL tubes (Thermo Scientific™, Rotor swing-out TH-641, catalog number: 12161690)

9. Dosage syringe 100 μL for Linomat (CAMAG, catalog number: 695.0014)

10. Semi-automatic sample dispenser (CAMAG, Linomat 5, catalog number: 022.7808)

11. ChemiDoc MP imaging system (Bio-Rad, model: ChemiDoc^TM^ XRS, catalog number: 1708265)

## Software and datasets

1. VisionCATs software (version 2.5, September 2019)

2. Image Lab^TM^ software (version 5.1, February 2014)

3. FIJI, Image J (version 1.53t, August 2022)

## Procedure

**A. Culture and membrane extraction**

The membrane extraction protocol described was adapted from [20], which is established from 5-days-old *Arabidopsis thaliana* seedlings grown in liquid conditions (refer to General notes 1 and 2). This membrane extraction protocol was performed with 7-day-old Col-0 ecotype seedlings.

1. Proceed with stratification of Col-0 seeds by addition of 1 mL of water in a 1.5 mL Eppendorf tube and incubation at 4 °C for 3 days with approximately 200 µL of seeds (corresponding to 120–160 mg of dry seeds).

2. Carry out sterilization of the seeds with a bleach solution for 25 min, followed by three 3 min washes with sterile MilliQ water.

3. Prepare a culture flask with 250 mL of half Murashige and Skoog (^1^/_2_ MS) liquid media and sterilize it at 110 °C for 30 min.

4. In sterile conditions and when the medium is back to room temperature, transfer the seeds to the flask under sterile conditions.

5. Place the flask on a table shaker at 110 rpm in a culture condition room (16 h light/8 h darkness at 22 °C) for 7 days.

6. Cool down a mortar, a pestle, and 50 mL Falcon tubes of membrane extraction buffer on ice.

7. Collect the plants, remove the excess liquid culture, and weigh their mass to harvest a minimum of 10 g of humid plant weight.

8. Rapidly deposit them in the mortar and grind them with a pestle by adding 2–3 volumes (v/w) of membrane extraction buffer + PMSF 1 mM (final concentration) prepared freshly.

9. When the plant solution is correctly crushed (when a homogeneous green solution is obtained), filter it with a gauze installed inside a funnel above a 50 mL Falcon.

10. Centrifuge the Falcon three times at 936 rcf for 10 min at 4 °C. The purpose of this step is to remove cell debris. Thus, at the end of each centrifugation, transfer the supernatant to a new Falcon and trash the Falcon containing the pellet.

11. In ultracentrifugation tubes of 12 mL, add to the bottom 2 mL of 38% sucrose solution. Very carefully, add around 9–10 mL of the plant homogenate above this cushion of sucrose, without disturbing the interface.

12. Equilibrate the ultracentrifugation rotor and start the ultracentrifugation at 122,253× g for 2 h at 4 °C.

13. Using a glass Pasteur pipette, harvest the yellow membrane phase present at the interface between the cushion of sucrose and the supernatant and place it in a 15 mL Falcon tube.

*Note: If the volume of membrane harvested is more than 9 mL, split the volume collected into two Falcons of 50 mL*

14. Fill the 15 mL Falcon to 12 mL with HEPES 50 mM solution and mix by inversion.

15. Transfer to new ultracentrifugation tubes of 12 mL.

16. Equilibrate the ultracentrifugation rotor and start the ultracentrifugation at 122,253× *g* for 2 h at 4 °C.

17. At the end of the ultracentrifugation, discard the supernatant carefully and resuspend the pellet with 0.2–1 mL of membrane resuspension buffer (depending on the size of the pellet). The microsomal fraction (total membrane fraction) is obtained and can be stored for a short time at 4 °C or at -80 °C for longer-term storage.

*Note: If there is more than one pellet for one condition, pool them.*

*Note: We recommend performing protein quantification with a Bicinchoninic Acid (BCA) Protein Assay kit at this step to estimate the amount of material in this microsomal fraction before directly performing phospholipid extraction by the addition of 725 µL of Phospholipid extraction “Kill” mix solution to the pellet (see step B1). We recommend obtaining a minimum of 2 mg/mL of proteins before proceeding to the phospholipid extraction.*

**B. Extraction of phospholipids of the previously obtained microsomal fraction**

The phospholipid extraction protocol described was adapted from [11], which was established from biological samples.

1. Add 725 μL of phospholipid extraction “Kill” mix and 150 μL of MilliQ water in the total membrane fraction sample and transfer it to a 2 mL Eppendorf.

2. Add 750 μL of chloroform.

3. Add 170 μL of 2 M HCl.

4. Shake vigorously by hand.

5. Vortex for 15 s.

6. Centrifuge at 1,500× *g* for 5 min

7. Remove the upper phase and transfer the lower phase to a new 2 mL Eppendorf tube.

*Note: Be careful at this step to limit the interactions and exchanges between the upper and lower phases*

8. Take 710 μL of the upper phase of the phospholipid wash mix and add it to the 2 mL Eppendorf tube that contains the lower phase of step B7.

9. Shake vigorously by hand.

10. Vortex for several seconds.

11. Centrifuge at 1,500× *g* for 3 min.

12. Remove the upper phase and transfer the lower phase to a new 2 mL Eppendorf tube.

13. Repeat step B8 two more times.

*Note: Be careful to delete the water drops in the upper phase at each step.*

14. This lipid extract can be stored at -20 °C in a 2 mL HPLC vial.

**C. Preparation of the migration solvent (under the fume hood)**

1. Prepare the PhosphoLIMBO mix.

a. Add 5 mL of chloroform to a 25 mL glassware.

b. Add 1.7 mL of methanol.

c. Add 4.2 mL of 2-propanol.

d. Add 1.3 mL of KCl at 0.25%.

e. Add 4 mL of methyl acetate.

f. Add 0.5 mL of triethylamine.

2. Mix the migration solvent by agitation.

3. Wash the CAMAG twin trough chamber with methanol.

4. Wash the CAMAG twin trough chamber a second time with some migration solvent (approximately 2 or 3 mL).

5. Deposit the entire volume of the migration solvent in the CAMAG twin trough chamber.

6. Close it immediately after.

7. Split the migration solvent equitably between the two gutters of the TLC developing chamber.

8. Wait for 25/30 min for the chamber to be saturated.

*Note: To limit evaporation, we advise adding a heavy object (like a glass bottle) to the CAMAG twin trough chamber during the entire saturation period and the migration step.*

**D. Loading of the lipid extract on an HPTLC plate**

As a control of migration, phospholipid standards are loaded on the plate, alone or in a mix. Please refer to General notes 3, 4, and 5.

1. Turn on the Linomat system to proceed to the loading of the plate.

2. Prepare the washing solution (chloroform/methanol 2:1 v/v).

3. Wash the syringe 3 times with the washing solution.

4. Prepare the loading plan on the HPTLC plate using the linomat software VisionCATs.

a. Plate format: 20 cm × 10 cm

b. Width: 200 mm

c. Height: 100 mm

d. Deposit height: 8.0 mm

e. First track position ≥ 30.0 mm

*Note: Be careful with side effects during migration; do not load lipid extract too close to the edge of the plate.*

5. Configure loading volumes.

6. Place the HPTLC Silica gel 60 F254 20 cm × 10 cm on the linomat stage.

7. Run the deposit procedure for the samples.

*Note: Do not forget to wash the syringe 3 times with the full volume of the syringe, e.g., 100 μL, between each sample using the washing solution.*

8. Let the plate dry on a vertical position for 5 min at room temperature.

**E. Migration procedure**

1. Place the HPTLC plate vertically in one of the two hinges of the CAMAG twin trough chamber and close it rapidly to limit the loss of the saturation solvent in the chamber.

*Note: The plate must be slightly immersed in the migration solvent and as close as possible to the inside face of the migration chamber.*

2. Wait without disturbing the migration (do not move the tank during the migration).

3. Wait until the migration solvent has reached the limit of the upper edge of the plate (until 2–3 mm from the upper edge).

*Note: For a 10-cm high plate, wait approximately 50–55 min.*

4. At the end of the migration, take out the plate of the tank and let it dry vertically under the fume hood.

**F. Visualization of lipid separation on the HPTLC plate**

The revelation of lipids is performed using primulin, which is a non-destructive lipid dye interacting in a nonspecific manner with the acyl chain of lipids (refer to General note 6).

1. Transfer the primulin solution into a glass recipient.

2. Carefully submerge the plate and take it out immediately.

*Note: Do so in such a way that the primulin solution covers the plate evenly.*

3. Let the plate dry for 10 min vertically in the dark.

4. Reveal the plate by UV light (302 nm) using the ChemiDoc system and the Image Lab^TM^ software.

## Data analysis

**Figure 1.**
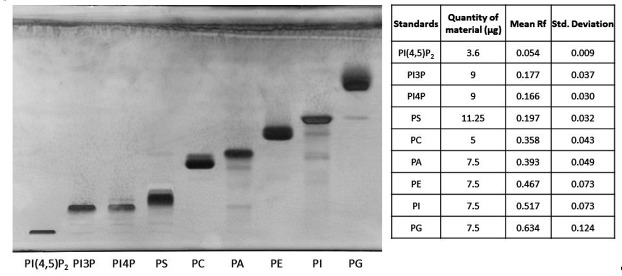
HPTLC separation of phospholipid standards using the PhosphoLIMBO protocol. Composition of the solvent mix: chloroform, methanol, 2-propanol, KCl 0.25%, methyl acetate, and triethylamine (30:10.2:25:7.8:24:3 v/v/v/v/v/v). Brain PI4,5P2: phosphatidylinositol-4,5-bisphosphate, 18:1. PI3P: phosphatidylinositol-3-phosphate, 18:1. PI4P: phosphatidylinositol-4-phosphate; soybean PS: phosphatidylserine; egg yolk PC: phosphatidylcholine; egg yolk PA: phosphatic acid; egg yolk PE: phosphatidylethanolamine; soybean PI: phosphatidylinositol; egg yolk PG: phosphatidylglycerol. Lipids were stained with primulin 2.5% [PBS 1×, acetone (50:50 v/v)]. N = 7 except for PI3P (n = 4).

**Figure 2.**
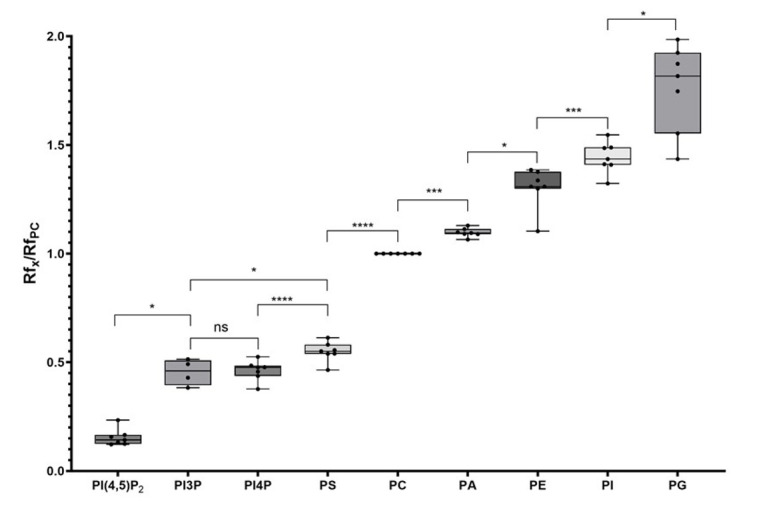
Quantification of the retention factor (Rf) of each phospholipid class. The Rf value for each phospholipid was calculated after migration on an HPTLC plate following the PhosphoLIMBO protocol. Rf values for each phospholipid are normalized against the Rf of PC (Rf_X_/Rf_PC_). N = 7 except for PI3P (n = 4). Statistical analysis was conducted by a parametric mixed-effects analysis: P-value = 0.0116 (*) PI4,5P_2_/PI3P; P-value = 0.0212 (*) PI3P/PS; P-value = 0.9992 (ns) PI3P/PI4P; P-value < 0.0001 (****) PI4P/PS; P-value < 0.0001 (****) PS/PC; P-value = 0.0002 (***) PC/PA; P-value = 0.0113 (*) PA/PE; P-value = 0.0015 (**) PE/PI; P-value = 0.0103 (*) PI/PG. The error bars indicate the minimum and maximum values. ns, non-significant.

**Figure 3.**
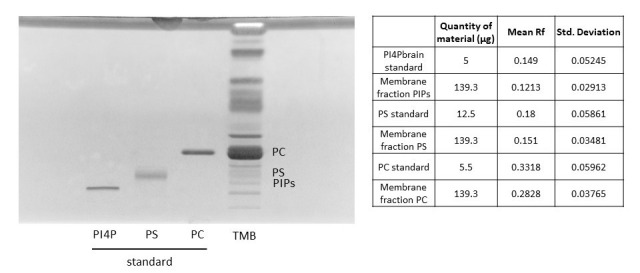
Migration of a microsomal fraction compared to phospholipid standards. HPTLC migration following the PhosphoLIMBO protocol. The microsomal fraction is a total membrane fraction (TMB) obtained after a sucrose cushion ultracentrifugation. Brain PI4P: phosphatidylinositol-4-phosphate; soybean PS: phosphatidylserine; egg yolk PC: phosphatidylcholine; and phospholipids were extracted from 7-day-old *Arabidopsis* seedlings TMB. Lipids were stained with primulin 2.5% [PBS 1×, acetone (50:50 v/v)]; n = 6.

Using the new solvent mix described in this protocol, we observed a scale distribution of the different phospholipid species on an HPTLC plate (Figure 1). This led us to call this new solvent mix the PhosphoLIMBO. All figures are associated with a respective table, which indicates the quantity loaded on the HPTLC plate. No data were excluded. By calculating the retention factor (Rf) value of each lipid species, we could extract the mean Rf out of 7 independent migrations (Figures 1 and 2). In PhosphoLIMBO, the phospholipid categories are gradually and significantly dissociated from each other. The Rf of PC, PA, PE, PI, and PG are 0.36, 0.39, 0.47, 0.52, and 0.63, respectively (Figure 2). These results show that PhosphoLIMBO is able to separate the different phospholipid species in a robust and reproducible manner (Figures 1 and 2). This hybrid method allows solving the co-migration of PA/PG [15] in Vitiello or PE/PI in Touchstone [14] (Figure 1, Extended Data 1 and 2). Importantly, PhosphoLIMBO allows the separation of the different species of anionic phospholipids and their separation from other phospholipid species also in membrane fraction (Figure 3). PIPs are significantly separated from PS (Rf = 0.17 for PIPs and 0.2 for PS), and PA is separated from PC (Rf = 0.35 for PC and 0.39 for PA) (Figures 1, 2, and 3). However, in this method, the PIPs phosphatidylinositol-3-phosphate (PI3P) and phosphatidylinositol-4-phosphate (PI4P) are not separated, as they display a similar Rf (0.177 for PI4P and 0.166 for PI3P) (Figure 2). The phosphatidylinositol-4,5-bisphosphate (PI4,5P_2_) is not mobilized by the PhosphoLIMBO solvent mix, as the Rf is close to 0 (Rf = 0.054, Figure 2). Additionally, we observed that two PI4P standards with different fatty acid composition (one is a mix of PI4P extracted from the brain, while the other one is a purified 18:1-PI4P species) migrate similarly, suggesting that the acyl-chain does not impact the migration, which probably relies only on the polar head (Figures 1 and 3). In this method, instead of calculating each individual Rf value, we propose reporting the Rf of the most abundant phospholipid detected, i.e., PC (RfPC). This approach improves reproducibility: for a given lipid, the Rf/RfPC value allows normalizing the Rf value of each phospholipid to correct for the small migration differences that could happen between individual replicates (Figures 1 and 2). This normalization allows the accurate identification of the different species of phospholipids by their Rf/RfPC ratio. The migration analysis could be performed by Fiji or ImageLab software to calculate the respective Rf of each lipid. We validated the efficient separation of phospholipid species, including the anionic phospholipids, by a parametric mixed-effects statistical test, as the values were paired and all expressed relatively to RfPC (Figure 2).

**Figure 4.**
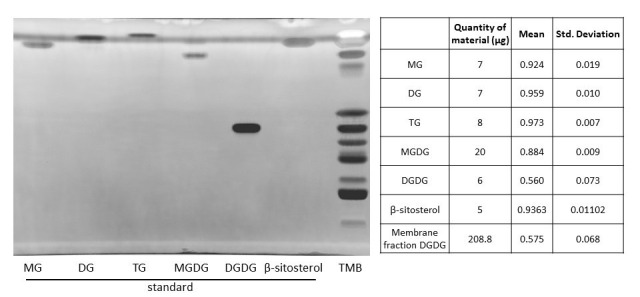
Migration of a microsomal fraction as compared to neutral lipid standards. HPTLC migration following the PhosphoLIMBO protocol. The microsomal fraction is a total membrane fraction (TMB) obtained after a sucrose cushion ultracentrifugation. 20:0 MG: monoacylglycerol; 18:1 DG: diacylglycerol; TG: triacylglycerol; plant MGDG: monogalactosyldiacylglycerol; plant DGDG: digalactosyldiacylglycerol; and plant β-sitosterol. Lipids were stained with primulin 2.5% [PBS 1×, acetone (50:50 v/v)]. N = 3 for MG, DG, TG, MGDG, DGDG, and β-sitosterol.

**Figure 5.**
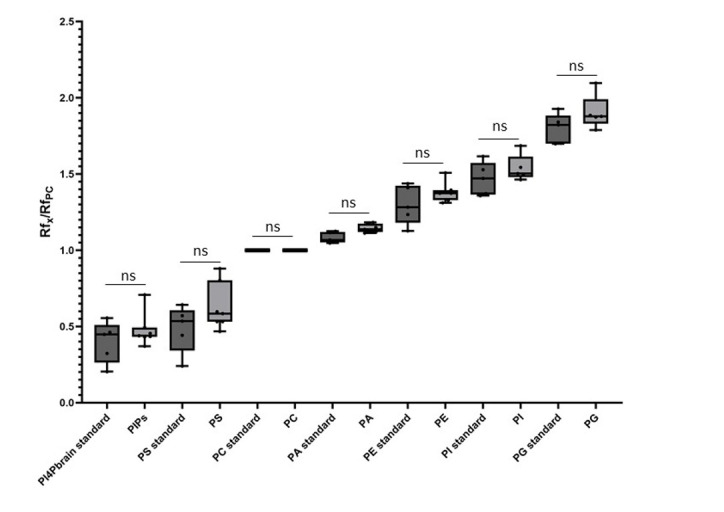
Comparison between the retention factor (Rf) of standard lipids and lipids contained in a microsomal fraction. The Rf value for each phospholipid is calculated after migration on the HPTLC plate following the PhosphoLIMBO protocol. Rf values for each phospholipid are normalized against the Rf of PC (Rf_X_/Rf_PC_). Statistical test is a parametric ANOVA test: P-value = 0.8523 (ns) PI4P standard/PIPs from TMB; P-value = 0.1605 (ns) PS standard/PS from TMB; P value > 0.9999 (ns) PC standard/PC from TMB; P-value = 0.9581 (ns) PA standard/PA from TMB; P-value = 0.7937 (ns) PE standard/PE from TMB; P-value = 0.8938 (ns) PI standard/PI from TMB; P-value = 0.4196 (ns) PG standard/PG from TMB. n = 5 for PI4P brain standard, PS standard, PC standard, PA standard, PE standard, PI standard, PG standard, PI, and PG. n = 7 for PIPs, PS, PC, PA, and PE. The error bars indicate the minimum and maximum values. ns, non-significant.

Additionally, using standards, we tested the migration of the neutral lipids monoacylglycerol (MG), diacylglycerol (DG), and triacylglycerol (TG), the galactolipids monogalactosylglycerol (MGDG) and digalactosylglycerol (DGDG), and the β-sitosterol, the main sterol in plants. Neutral MG, DG, and TG migrated to the front with a Rf value close to 1 (Figure 4). Galactolipids MGDG and DGDG displayed an intermediate migration profile between neutral lipids and galactolipids, with DGDG migrating close to PI (Figure 4). MGDG and DGDG had an Rf of 0.88 and 0.56, respectively (Figure 4). For some unknown reason, PG and DGDG displayed a larger range of migration as compared to other lipids tested in this protocol (Rf = 0.63 ± 0.12 for PG and Rf = 0.56 ± 0.073 for DGDG). However, PG and DGDG were significantly separated using the PhosphoLIMBO solvent mix. The major sterol in plants, the β-sitosterol, migrated as a neutral lipid at the front of the plate with an Rf of 0.94 (Figure 4). Finally, we tested whether the lipids extracted from the biological matrix we used in this protocol, i.e., microsomal fraction, migrated in a similar way (tested by a parametric ANOVA test as the values are unpaired) as the individual purified standards we used to identify the endogenous lipids (Figure 5). Thus, there is no matrix effect during migration.

One important aspect of this protocol is the nature and quantity of the starting material. Here, we produced a microsomal fraction because we were interested in membrane lipids; this provided an enrichment of the total membrane pool. As a guideline, we advise starting with more than 4–5 g of fresh material to produce a microsomal fraction. However, lipid extraction can be run on whole plant seedlings without any prior membrane purification. In this case, lipid concentration might be lower than if purifying a microsomal fraction beforehand, and thus the signals obtained might be less intense. After obtaining a microsomal fraction, we always quantify the protein content to obtain a value expressed in μg/μL. This value helps us to evaluate the volume of lipid extract to load on the HPTLC plate. After lipid extraction, no proteins are left in the extract, but if we virtually consider that all proteins remain in the lipid extract, then we advise loading on the HPTLC plate the equivalent of around 150 µg of proteins to be able to visualize anionic phospholipids such as PS and PIPs. A lower amount of equivalent proteins could be used to visualize the more abundant class of phospholipids, such as PC, PE, PI, and PG.

Our protocol can be coupled to quantification by gas chromatography coupled with mass spectrometry (GC–MS). To do that, the lipid bands are scratched from HPTLC plates, transmethylated, and injected in GC–MS (a detailed protocol is given in [19,20]. One advantage of this add-on method is the identification of the fatty acid composition in the pool of each lipid class analyzed. However, only abundant phospholipids such as PC, PE, and PI can be measured by GC–MS. Quantification of PS by HPTLC coupled with GC–MS might be too close to the detection limit, and PIPs are not quantifiable by GC–MS due to their low abundance. Another possible method is to measure the quantity of each phospholipid class by densitometry, either with a dedicated scanner or a classical imager equipped with a UV lamp to read the primulin staining. Quantification of lipid bands can be performed by integration of the peaks using the plot profile function of Image J. Each quantity of lipid standards or lipid extract from a biological sample (expressed in µg of proteins detected in the sample prior to lipid extraction) we deposited on HPTLC plates are indicated on the left side of Figures 1, 3, and 4 and in Supplemental information 1 and 2.

As a general advice, experiments with biological samples should be replicated at least 3 times, and accompanied on side tracks with phospholipid standards adapted for the lipid of interest. Moreover, due to the instability of anionic phospholipids after extraction, we highly recommended working with fresh samples (avoid freezing of the plant material) and loading the lipids on the HPTLC plate on the same day of their extraction.

In conclusion, the PhosphoLIMBO protocol is an advantageous method to efficiently separate all phospholipid classes, including anionic phospholipids. Compared with previously published protocols such as Vitiello and Touchstone, this mixed protocol allows an easy and cost-effective method that can be combined with densitometry or GC–MS quantification in an accessible and robust manner (Table 1).

**Table 1.** Recipes of the solvent migration mixes used for phospholipid separation by HPTLC

	Vitiello solvent [15]	Touchstone solvent [14]	PhosphoLIMBO solvent
Chloroform	26.6%	34.1%	30.3%
Methanol	10.6%	10.2%	10.4%
2-propanol	26.6%	28.4%	24.2%
Methyl acetate	26.6%	-	24.2%
Triethylamine	-	20.4%	3%
KCl (0.25%)	9.5%	6.8%	7.9%

## Validation of protocol

The PhosphoLIMBO protocol was used in previously published studies [3,19]. The figures of this article further validate the protocol by quantifying the Rf value, confirming the separation of all phospholipid classes by statistical tests, and testing its efficiency in biological samples.

## General notes and troubleshooting

**General notes**

1. The liquid culture could be replaced by growing *Arabidopsis* seedlings on vertical ½ MS plates. However, be careful to have enough material (see General note 2).

2. In terms of quantity, we performed the total membrane extraction from 4–5 g of fresh seedlings of 7-day-old specimens.

3. We strongly recommend performing HPTLC with phospholipid extracts freshly prepared due to the instability of anionic phospholipids, even if the lipid extract was kept at -20 °C.

4. The loading step could be done by hand, but this could result in a lower quality separation.

5. For the replicates, we advise using HPTLC silica plates from the same batch to avoid inter-batch variability.

6. The quantity loaded on HPTLC plates needs to be sufficient to observe PIPs and PS. We recommended loading between 5 and 12 μg of standards. As for biological samples, we recommend loading around 150 μg of equivalent protein (see Data analysis for more explanations).

7. The classical primulin revelation (80% of acetone) is too weak to reveal the PIPs. We advise using the primulin solution as described in the Recipes section (with PBS). However, it is very important that the HPTLC plate is immersed only very briefly in this solution to avoid any undesirable crackling effect of the silica. Alternatively, spraying the primulin solution on the HPTLC plate is also possible.

**Troubleshooting**

**Table:** 

Troubleshooting	Cause	Solution
Pathogen contamination in the plant culture flask	Issue during the sterilization procedure (of the liquid ½ MS medium or the seeds)	Repeat a new culture. Grow the seeds on vertical ½ MS solid plates instead of liquid culture.
No membrane phase (yellow phase) after the first ultracentrifugation	Limited amount of biological material	Repeat a new plant growth culture with a higher number of seeds (120–160 mg of dry seeds).
	The membranes were not intact after extraction	Prepare a new membrane extraction buffer and HEPES solution with a pH of 7.5.
	Issue during membrane extraction	Be aware to conduct steps A6–13 in cold conditions with pH 7.5 HEPES solution (the pH must be adjusted only with potassium hydroxide). For step A11, manipulate the sucrose cushion and the plant homogenate in a very careful way, not to disturb the interface.
	Excess of membrane extraction buffer	Be aware to add the appropriate volume of extraction buffer.
The membrane quantity (relative to protein concentration) is not sufficient for phospholipid extraction	Some proteases are still functional	Repeat the culture and membrane extraction. Add PMSF to the membrane extraction buffer. Add PMSF and cocktail protease inhibitor to the membrane resuspension buffer extemporaneously.
	Limited amount of biological material	Repeat a new plant growth culture with a higher number of seeds (120–160 mg of dry seeds).
	The membrane extraction sample is too old	Repeat a new culture and perform membrane extraction, phospholipid extraction, and HPTLC within the same day.
There are no bands corresponding to PIPs in the biological sample	Limited amount of biological material	Repeat a new plant growth culture with a higher number of seeds (120–160 mg of dry seeds)
	Issue during the anionic phospholipid extraction	Repeat the anionic phospholipid extraction step and be aware to prepare correctly the phospholipid extraction buffer and the phospholipid wash mix; HCl is critical to correctly extract anionic phospholipids.
	The membrane extraction sample is too old	Repeat a new culture and perform membrane extraction, phospholipid extraction, and HPTLC extemporaneously.
	Issue during the primulin staining protocol	The classical primulin revelation (80% of acetone) is inadequate to reveal the presence of PIPs. We advise using the primulin solution prepared with PBS, as described in the Recipes section.
The retention factor is the same for several phospholipids (standards and biological samples)	The HPTLC migration is not efficient	Let the migration of the PhosphoLIMBO solvent mix move to the top of the plate
	The plate used is not adapted	The migration must be done on HPTLC Silica gel 60 F254
	Issue during the preparation of the PhosphoLIMBO migration solvent	Repeat the migration and carefully prepare the PhosphoLIMBO migration solvent mix extemporaneously.
The HPTLC silica plate cracked during and/or after the primulin staining	Issue during the primulin staining protocol	We advise using the primulin solution with PBS as described in the Recipes section. However, it is very important that the HPTLC plate is only immersed very briefly in the primulin solution to avoid any undesirable crackling effect of the silica. Alternatively, spraying this primulin solution on the surface of the HPTLC plate is also possible.

## Supplementary information

The following supporting information can be downloaded here:

1. Supplemental information 1. HPTLC separation of phospholipids, including anionic phospholipids, using the Touchstone solvent mix.

2. Supplemental information 2. HPTLC separation of phospholipids, including anionic phospholipids, using the Vitiello solvent mix.
